# Targeting the key cholesterol biosynthesis enzyme squalene monooxygenasefor cancer therapy

**DOI:** 10.3389/fonc.2022.938502

**Published:** 2022-08-24

**Authors:** Yuheng Zou, Hongying Zhang, Feng Bi, Qiulin Tang, Huanji Xu

**Affiliations:** ^1^ Department of Medical Oncology, Cancer Center and Laboratory of Molecular Targeted Therapy in Oncology, West China Hospital, Sichuan University, Chengdu, China; ^2^ Laboratory of Oncogene, West China Hospital, Sichuan University, Chengdu, China

**Keywords:** cancer treatment, cell proliferation and migration, cholesterol metabolism, drug target, squalene epoxidase

## Abstract

Cholesterol metabolism is often dysregulated in cancer. Squalene monooxygenase (SQLE) is the second rate-limiting enzyme involved in cholesterol synthesis. Since the discovery of SQLE dysregulation in cancer, compelling evidence has indicated that SQLE plays a vital role in cancer initiation and progression and is a promising therapeutic target for cancer treatment. In this review, we provide an overview of the role and regulation of SQLE in cancer and summarize the updates of antitumor therapy targeting SQLE.

## Highlights

1. Squalene monooxygenase (SQLE) as the second rate-limiting enzyme involved in cholesterol synthesis, is dysregulated in tumors.
2. The dysregulation of SQLE is associated with poor prognosis and resistance to some therapies (radiation, hormone deprivation therapy).
3. SQLE promotes tumor growth, while inhibition SQLE can restrain tumor growth across various types of cancer. Targeting SQLE may be a potential direction for novel anti-cancer therapy.

## Introduction

Squalene monooxygenase (SQLE) catalyzes the oxidation of squalene to (S)-2,3-epoxysqualene (1). For quite a long time, it has been investigated as an anti-fungal target because the epoxysqualene derivative lanosterol is a component of the fungal membrane (2, 3). In human cells, the gene encoding SQLE is located at chromosome region 8q 24.1 (4). SQLE is a direct target of sterol regulatory element binding protein-2 (SREBP2), a transcription factor that regulates genes involved in cholesterol biosynthesis and homeostasis in a cholesterol-dependent manner; SQLE protein also contains a cholesterol sensing domain that can regulate proteasomal degradation of SQLE (5). Therefore, like 3-hydroxy-3-methylglutaryl-CoA reductase (HMGCR), SQLE activity is also precisely regulated by intracellular cholesterol level in the form of feedback, which makes it a second rate-limiting step in cholesterol synthesis (2, 3).

Numerous studies have revealed that the deregulation of SQLE results in cholesterol metabolism disorder and is associated with many diseases, including Alzheimer’s disease, hypercholesterolemia, stroke, and cancer (6). Tumor cells exhibit a high requirement for energy and materials to meet the demands for rapid tumor growth. SQLE is crucial for cancer cells to meet the requirement for cholesterol. A high abundance of SQLE has been identified in multiple cancer types and has emerged as a hot topic in the field of cancer treatment (7).

In this review, we discuss the regulation of SQLE, its roles and its clinical relevance in cancers. Cholesterol metabolism centered on SQLE is described in brief. Finally, the latest developments in antitumor therapies targeting SQLE are summarized.

## Brief overview of cholesterol metabolism

Cholesterol plays manifold roles in normal cells and tumor regression. As an essential lipid component of the mammalian cell membrane, cholesterol is vital for cell survival and proliferation. By maintaining the stability of lipid rafts, cholesterol can coordinate the signal transduction of multiple membrane receptors. In addition, cholesterol can act as a signaling molecule to directly regulate the activation of signaling pathways in cancer cells. The cellular cholesterol level is determined by a complex network, mainly including cholesterol biosynthesis, uptake, export, and esterification (7).

**Figure 1:**
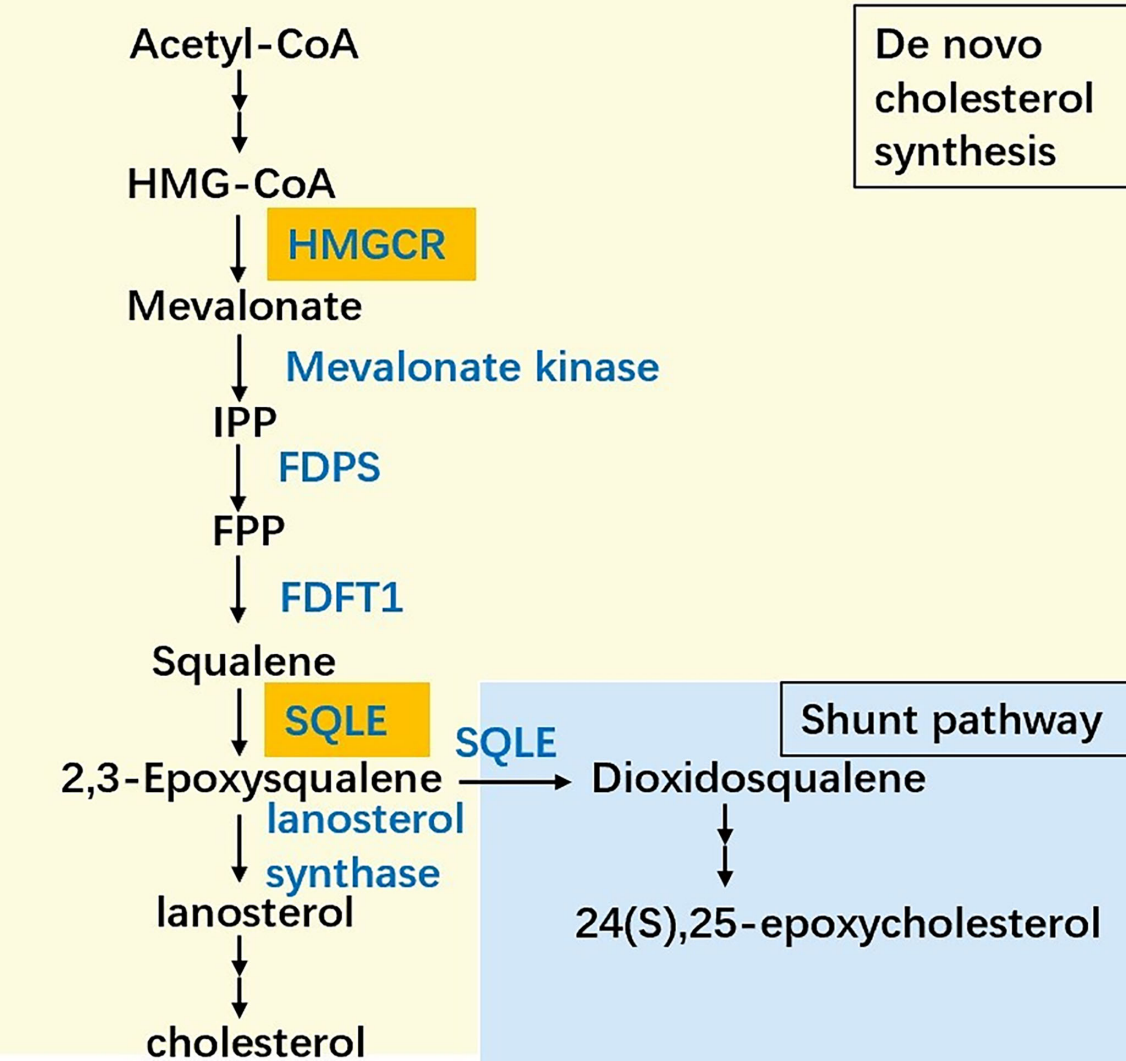
The simplified scheme of cholesterol biosynthesis. The biosynthesis pathway converts acetyl-CoA into cholesterol more than 20 enzymatic reactions, among which HMG-CoA reductase (HMGCR) and squalene epoxidase (SQLE) are the two key speed-limiting enzymes. Besides, SQLE can divert 2,3-epoxysqualene into dioxidosqualene. The end product of the shunt pathway, 24(S),25-epoxycholesterol can regulate the cholesterol metabolism in turn. IPP: Isopentenyl-PP, FPP: Farnesyl-PP, FDPS: Farnesyl-diphosphate farnesyltransferase 1, FDFT1: Farnesyl-diphosphate farnesyltransferase 1.

Almost all mammalian cells can *de novo* synthesize cholesterol from acetyl-CoA to cholesterol through more than 20 enzymatic reactions, including the mevalonate (MVA) pathway, squalene biosynthesis and subsequent reactions (**Figure 1**). In the cholesterol synthesis pathway, two rate-limiting enzymes, HMGCR and SQLE, are essential players (8). SQLE is responsible for the first oxygenation step in cholesterol synthesis, which converts squalene to 2,3-epoxysqualene (9). SQLE can also divert 2,3-epoxysqualene into 2,3(S),22(S),23-dioxidosqualene, especially when the activity of lanosterol synthase, the enzyme that converts 2,3-epoxysqualene to lanosterol, is low (10). The end product of this shunt pathway, 24(S),25-epoxycholesterol, is the ligand for liver X receptors, which can upregulate ATP-binding cassette transporter A1 (ABCA1) levels to promote cholesterol efflux. Loss of 24(S),25-epoxycholesterol can induce acute cholesterol synthesis by increasing HMGCR expression processed by SREBP2 (11). Collectively, reactions catalyzed by SQLE are crucial for cholesterol metabolism.

## SQLE is highly expressed in cancer

Cancer is a complex disease involving the dysregulation of cell proliferation, energy metabolism, angiogenesis, and immune surveillance (12). Given the characteristics of tumors and the function of cholesterol, cholesterol metabolic reprogramming is a milestone in cancer development. In recent years, SQLE has garnered increasing attention for its association with cancer. Various cancers have been found to exhibit high levels of SQLE protein/mRNA or have SQLE copy number alterations (13).

As an oncogene for breast cancer, gene amplification and overexpression of SQLE have been reported in cancer tissues/ductal carcinoma *in situ* tissues compared with normal tissues (14–17). Race expression differences have been reported: the expression of SQLE in African-Americans is higher than that in Caucasians in luminal A breast tumors and basal-like breast cancers (18, 19). In prostate cancer, the expression of SQLE is upregulated during the progression of cancer (20, 21). In pancreatic cancer, SQLE is upregulated and sqle gains in tumor tissues. In colorectal cancer, SQLE is upregulated in tumor tissues compared with normal tissues, but SQLE in stages I, II, and III is higher than that in stage IV (22, 23). Similarly, SQLE is upregulated in nasopharyngeal cancer (13), head and neck squamous cell carcinoma (24), leukemia (25), hepatocellular cancer (26–28), and squamous lung cancer (29, 30).

## The mechanisms that regulate SQLE expression in cancer

**Figure 2:**
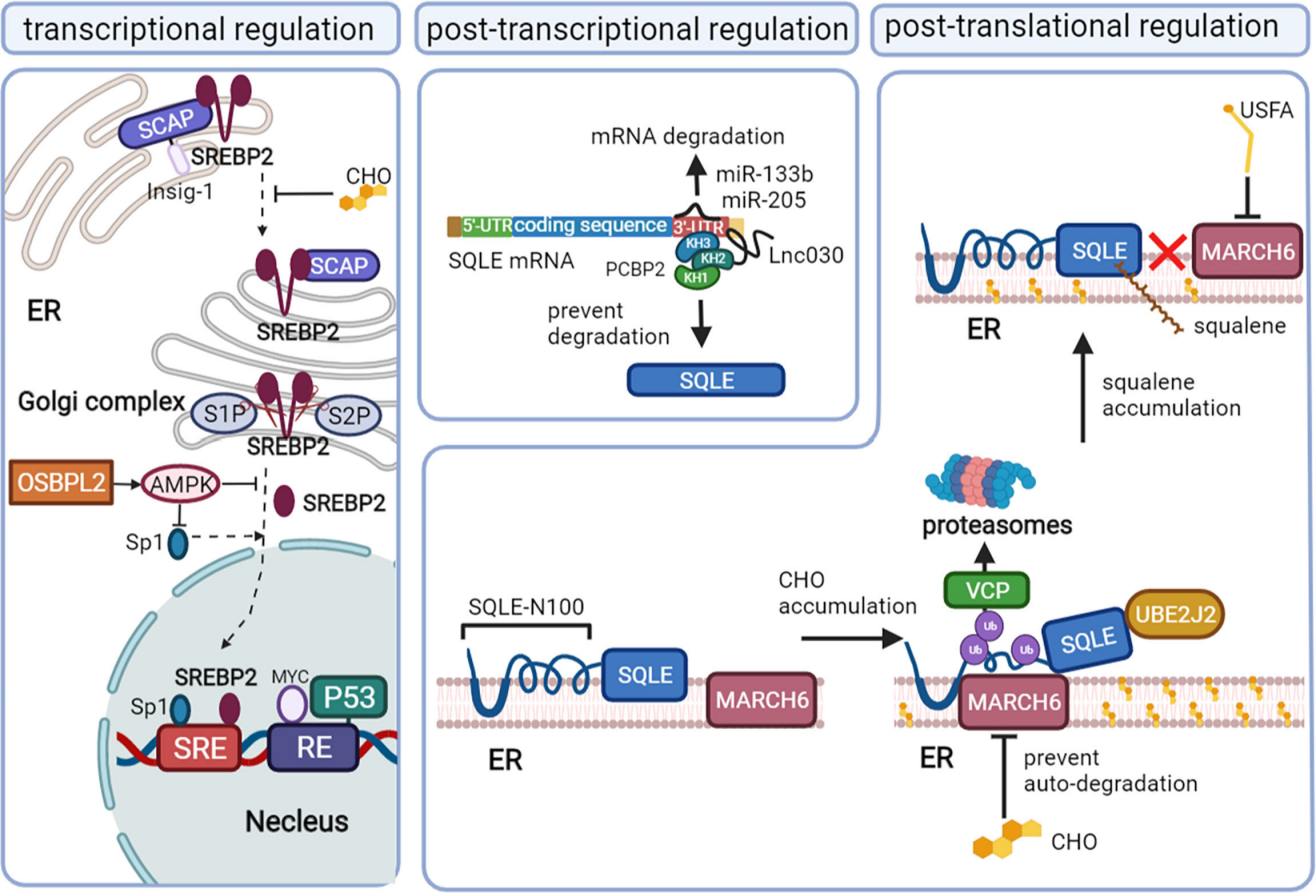
The mechanisms that regulate SQLE expression in cancer. Squalene monooxygenase (SQLE) can be regulated at the transcriptional level mainly *via* sterol regulatory element-binding protein 2 (SREBP2), the posttranscriptional level *via* microRNA (miRNA)/long noncoding RNA (lncRNA), and the posttranslational level *via* cholesterol feedback regulation. Mature SREBP2 promotes the transcription of SQLE by binding to the sterol-regulatory elements (SREs) of sqle (the gene encoding SQLE). When the cholesterol content in the endoplasmic reticulum membrane decreases, the SREBP2/SCAP complex dissociates from Insig-1 on the endoplasmic reticulum (ER) and is convoyed to the Golgi complex, where SREBP2 is cleaved by site-1 protease (S1P) and site-2 protease (S2P) and releases the mature form in the Golgi complex. Oxysterol binding protein like 2 (OSBPL2) deficiency can promote SREBP2 and specificity protein (Sp1) activation by inhibiting AMPK. P53 and MYC also regulate SQLE at the transcriptional level. MiR-133b and miR-205 promote SQLE mRNA degradation, while Lnc030 prevents SQLE mRNA degradation by forming a poly(rC) binding protein 2 (PCBP2)/Lnc030/SQLE 3’-UTR complex. Cholesterol (CHO) feedback regulation occurs *via* ubiquitin-proteasomal degradation of SQLE, which requires ubiquitin-conjugating enzyme E2 J2 (UBE2J2), membrane-associated RING finger 6 (MARCH6) and conformational change of SQLE N-100. Valosin-containing protein (VCP) extracts SQLE from the ER to proteasomes. Squalene and unsaturated fatty acids (USFAs) can disrupt the interaction between SQLE N-100 and MARCH6 by regulating SQLE and MARCH6, respectively, and suppress ubiquitin-proteasomal degradation.

SQLE, as the second rate-limiting enzyme of cholesterol synthesis, is regulated exquisitely by a complex network, including the transcription program, posttranscriptional program, and posttranslational program. Recent studies have reported novel mechanisms that result in the high expression of SQLE in cancer (**Figure 2**).

### Transcriptional regulation

The transcription factor SREBP2 directly regulates the mRNA levels of enzymes involved in sterol metabolism, including HMGCR, LDL, and SQLE, by binding the sterol-regulatory element (SRE) sequence in the promoters of target genes (31–33). The maturation of SREBP2 and its translocation depend on the intracellular cholesterol level. When the cholesterol level in the endoplasmic reticulum (ER) membrane increases, SREBP2 is retained at the ER due to its binding partner SREBP2 cleavage-activating protein (SCAP) interacting with an ER membrane anchor protein insulin-induced gene (Insig-1) protein. When the ER membrane cholesterol level decreases, SCAP undergoes a conformational change and dissociates from the Insig-1 protein and then convoys SREBP2 from the ER to the Golgi complex, where SREBP2 is proteolytically cleaved by site-1 protease (S1P) and site-2 protease (S2P) (34). The N-terminal domain of SREBP2 then enters the nucleus, binding with SRE to upregulate the mRNA level of SQLE (32).

In addition, specificity protein 1 (Sp1) and nuclear factor Y (NF-Y) may be required to coregulate SQLE levels with SREBP2, as evidenced by their binding sites on the SQLE gene (32, 33). The deficiency of oxysterol binding protein like 2 (OSBPL2) can also activate SREBP2 and Sp1, coregulating the expression of SQLE by inhibiting the adenosine 5’-monophosphate (AMP)-activated protein kinase (AMPK) pathway (35).

The gain of oncogenes and the loss of cancer suppressor genes are also involved in the transcriptional regulation of SQLE independent of SREBP2. MYC upregulates the transcription program of SQLE by binding the response element 1 (RE1) of the SQLE gene in cancer (36, 37). The cancer suppressor protein p53 suppresses the transcription of SQLE by directly binding to the RE of the SQLE gene in hepatocellular carcinoma cells. Loss of p53 can augment the expression of SQLE, even under normal or elevated ER membrane cholesterol levels (38).

### Post transcriptional regulation

Long noncoding RNA (lncRNA) and microRNA (miRNA) can interact with SQLE mRNA and then influence the stability of SQLE mRNA. In breast cancer, lnc030 is highly expressed to stabilize SQLE mRNA, especially in cancer stem cells. The stabilization function of lnc030 requires poly(rC) binding protein 2 (PCBP2), the 3’ untranslated region (3’UTR) of SQLE mRNA and lnc030 to form a complex. Lnc030 interacts with the K homology domains 2 of PCBP2, while the 3’UTR of SQLE mRNA binds to K homology domains 3 of PCBP2 (39).

MiR-133b is downregulated in esophageal squamous cell carcinoma and can bind directly to the 3’UTR of SQLE mRNA. *In vitro*, ectopic expression of miR-133b can decrease the mRNA and protein levels of SQLE (40). Another miRNA, miR-205, has also been reported to suppress the expression of SQLE by binding to the 3′-UTR of SQLE mRNA in progressive prostate cancer, where its expression is decreased (41).

### Post translational regulation

Cholesterol, the end product of SQLE, plays an important role in regulating SQLE stability mainly *via* the cholesterol-membrane-associated RING finger 6 (MARCH6)-proteasomal degradation axis (42–44). The first 100 amino acids of SQLE (SQLE N-100), which can sense the cholesterol level in the cytoplasm (45, 46), can be attached to the ER membrane by a re-entrant loop. The Gln62–Leu73-sequence can form the amphipathic helix buried reversibly in the membrane, which is required for cholesterol-dependent degradation (47, 48). The accumulation of intracellular cholesterol thickens the anchoring of SQLE to the ER membrane, leading to the exposure of the hydrophobic core to the aqueous phase and triggering conformational changes in the re-entrant loop and amphipathic helix. Proteasomal degradation would be absent if these changes were disrupted (45).

The ubiquitin-proteasomal degradation system also requires the E2 ubiquitin-conjugating enzyme J2 (UBE2J2) and the E3 ubiquitin ligase MARCH6, unidentified deubiquitinases (48–51). MARCH6 ubiquitinates serine residues near the flanking amphipathic helix of SQLE deformed by excess cholesterol (51, 52). UBE2J2 is an important partner of MARCH6 in the cholesterol-stimulated degradation of SQLE. Valosin-containing protein (VCP), known to mediate the degradation of ubiquitinated endoplasmic reticulum-associated degradation (ERAD) substrates (53), is recruited downstream of ubiquitination, extracting SQLE from the ER and allowing proteasomal degradation (48). Excess cholesterol can stabilize MARCH6 by inhibiting its ubiquitination-proteasomal degradation, which in turn stimulates the degradation of SQLE (54).

Proteasomal degradation *via* MARCH6-VCP can also partially degrade SQLE from the N-terminus in a distinct ubiquitination pathway independent of cholesterol regulation, converting full-length SQLE to trunSQLE in various cell types. The enzymatic activity of trunSQLE is cholesterol-resistant, preventing the complete ablation of SQLE function under excess cholesterol levels (46). TrunSQLE might confer a supplemental route of cholesterol metabolism in pathophysiological contexts, especially in cancer.

Not only cholesterol but also squalene, the substrate of SQLE, can regulate SQLE expression by posttranslational modification. Squalene can directly bind to the SQLE N-100 domain, which is also squalene-sensitive, thereby inhibiting the interactions between MARCH6 and the SQLE N-100 domain. Thus, the accumulation of squalene can stabilize SQLE by preventing its proteasomal degradation (55–57). Unsaturated fatty acids (USFA) can also stabilize SQLE by blocking ubiquitination (58). The mechanism of USFA-mediated stabilization appears to occur through regulating MARCH6. In breast cancer, upregulation of the transmembrane microprotein cancer-associated small integral membrane open reading frame 1 (CASIMO1) increases the level of SQLE *via* interaction with SQLE proteins (59). The interaction of CASIMO1 and SQLE proteins may prevent the degradation of SQLE.

In summary, the cholesterol-dependent feedback regulation of SQLE *via* SREBP2 transcriptional regulation and ubiquitin-proteasomal degradation are the main mechanisms of SQLE regulation in various cells. In tumor tissues, the activation of SREBP2 leads to the high expression of SQLE. In addition, the activation of oncogenes, the loss of cancer suppressor genes, and the dysregulation of some lncRNAs, miRNAs and cancer-associated proteins also contribute to the upregulation of SQLE. The dysregulation of SQLE may be the result of multiple events in cancer, while a specific mechanism might prevail in a specific subset of tumor cells.

## Tumor promotion by high SQLE expression

As mentioned above, cholesterol metabolic reprogramming is a hallmark in various cancer types and has been confirmed to promote tumor initiation and progression (7). As the key enzyme in the steps of cholesterol synthesis, the activity of SQLE determines the abundance of cholesterol and cholesterol derivatives in cancer (13, 39). SQLE can promote tumor growth *via* cholesterol/cholesteryl ester accumulation and the subsequent activation of multiple oncogenic pathways, such as PI3K/AKT signaling (13, 39). Some studies emphasized the role of cholesteryl ester rather than cholesterol in promoting tumor cell growth, as evidenced by the fact that the inhibition or knockdown of sterol O-acyltransferase (SOAT1/2) abolished the growth-promoting effect of SQLE (13, 28).

In addition to cholesterol-dependent effects, SQLE can also activate AKT by silencing PTEN in NAFLD-induced hepatocellular carcinoma (HCC). NADPH is required for the conversion of squalene to 2,3-epoxysqualene catalyzed by SQLE. The exhaustion of NADPH by SQLE induces oxidative stress, leading to the epigenetic modification of PTEN by the activation of DNA methyltransferase 3A (DNMT3A). Loss of PTEN activates AKT/mTOR pathways, subsequently activating the expression of SOAT1/2 and contributing to the accumulation of cholesteryl ester and NAFLD-induced HCC (28). Cholesteryl ester accumulation driven by the PTEN/PI3K/AKT/mTOR pathway in prostate cancer cells and subsequent activation of SOAT1 were reported previously (60). Collectively, these staggered cascade reactions amplify the cancer-promoting effect of SQLE.

In multiple cancer types, extracellular signal-regulated kinase (ERK), a crucial oncogenic signaling molecule, has been reported to be regulated by SQLE (26, 59, 61). Recently, HE L et al. revealed the underlying mechanism of ERK activation by SQLE. In colorectal cancer, knockdown of SQLE reduced calcitriol, the active metabolite of VitD3, leading to a reduction in cytochrome P450 family 24 subfamily A member 1 (CYP24A1) levels, which suppressed the phosphorylation and activation of ERK (22).

In addition to cell intrinsic effects, SQLE partially promotes tumor growth *via* host-microbiota interaction. The gut dysbiosis and altered the metabolism of the gut metabolism caused by elevated expression of SQLE, triggered the gut barrier defects and pro- inflammatory factors. Inquiringly, Transplantation of fecal bacteria from Sqle transgenic mice to germ-free mice can impair gut barrier function and stimulate cell proliferation, compared with fecal bacteria from control mice (62).

**Figure 3:**
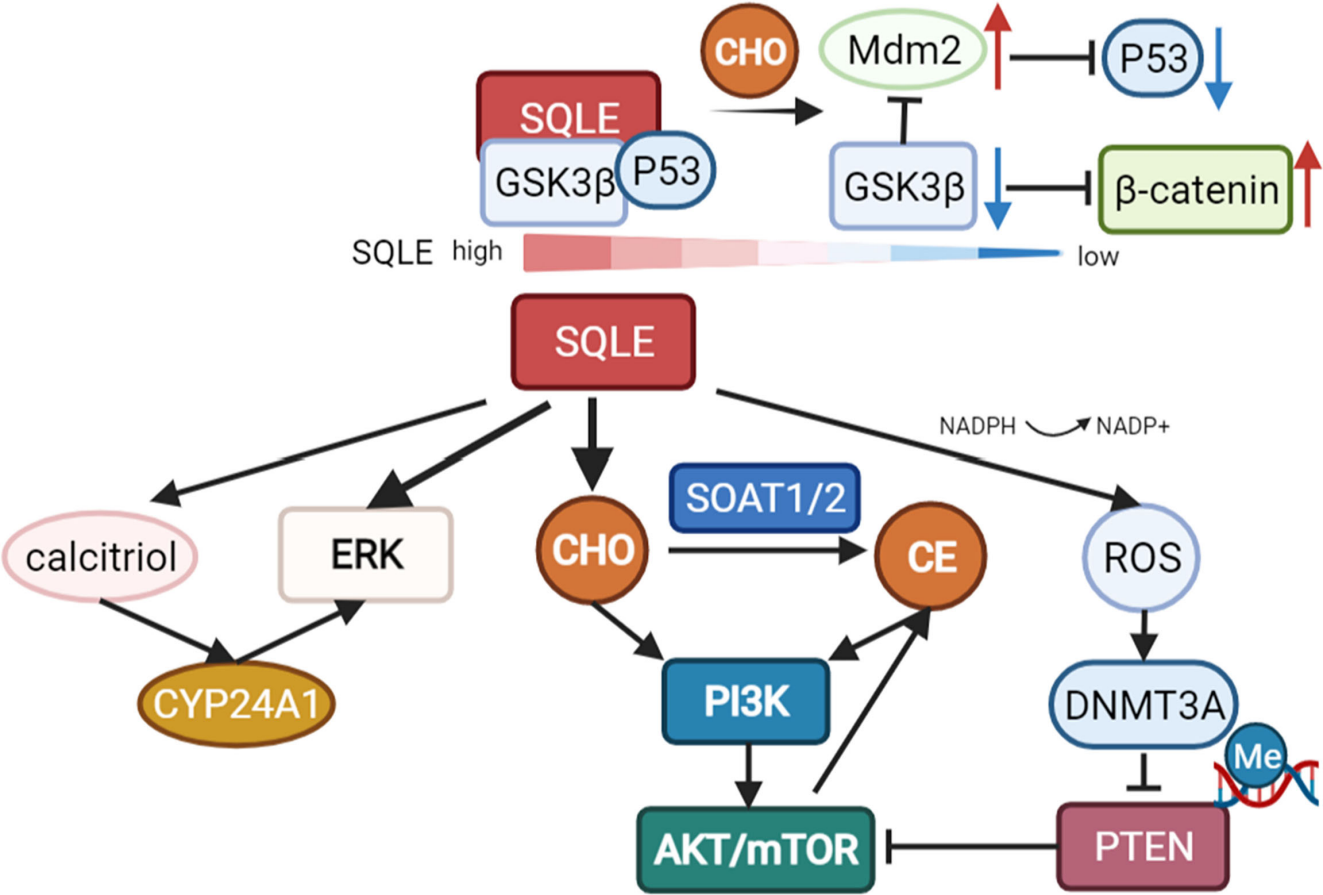
Pathways regulated by SQLE. SQLE can activate multiple oncogenic pathways, such as PI3K/AKT/mTOR signaling and the ERK pathway, *via* cholesterol/cholesteryl ester accumulation. Knockdown of SQLE can also reduce calcitriol, the active metabolite of VitD3, leading to a reduction in cytochrome P450 family 24 subfamily A member 1 (CYP24A1) levels, which suppresses the phosphorylation and activation of ERK. The exhaustion of NADPH during the conversion of squalene to 2,3-epoxysqualene by SQLE can induce oxidative stress and subsequently activate DNA methyltransferase 3A (DNMT3A), leading to the epigenetic silencing of PTEN. Loss of PTEN activates AKT/mTOR pathways, contributing to SOAT-mediated cholesteryl ester accumulation and NAFLD-induced HCC. However, there is also a report that depletion of SQLE can dissociate GSK3β and p53 and upregulate Mdm2, promoting the degradation of p53 and activation of β-catenin in colorectal cancer.

However, there is a report that cholesterol accumulation in colorectal cancer can downregulate SQLE and in turn promote tumor metastasis by activating epithelial-mesenchymal transition (EMT)-related pathways. Depletion of SQLE dissociates glycogen synthase 3b (GSK3β) and p53 and upregulates Mdm2, subsequently promoting the degradation of p53 and activation of β-catenin. Consequently, E-cadherin is downregulated, indicating the induction of EMT (23). However, many other studies revealed that EMT factors are upregulated with increased levels of SQLE in cancers, including colorectal cancer (63), pancreatic adenocarcinoma (64), and esophageal squamous cell carcinoma (40). These studies indicated that SQLE is associated with EMT but need to be further studied in certain tumor types (**Figure 3**).

## Clinical relevance of SQLE in cancer

SQLE can promote cell proliferation (24) and cell migration (65), adjust the cell cycle, and repress cell apoptosis (13, 28), contributing to the different phenotypes of tumors. Deregulated SQLE is associated with tumor aggressiveness and therapy resistance, indicating the poor prognosis of cancers, as reported in previous studies (**Table 1**).

**Table 1:** Clinical relevance of SQLE in cancer.

Cancer type	Clinicopathological variable relevance	Therapy response	Prognosis
Prostate cancer	Positive correlation with Gleason score (71, 72) Higher SQLE in high grade acinar cancer compared with ductal carcinoma of the prostate (73)	SQLE overexpression involved in the resistance to ADT (castration) (78)	Higher SQLE associated with metastasis, poor PFS, OS (72, 74)
Breast cancer	SQLE overexpression associated with larger tumor size, advanced TNM stage, HER2(+) status and lymph node metastasis (16, 17)	SQLE overexpression associated with the resistance to EDT (letrozole) (15)	Higher SQLE associated with poor OS, tumor recurrence (14, 16, 79, 80)
Hepatocellular cancer	SQLE overexpression is associated with advanced TNM stage, a-fetoprotein elevation (82)	No relevant research	Higher SQLE associated with poor prognosis (82) Independent prediction of poor prognosis (28)
Pancreatic cancer	No relevant research	SQLE associated with radiation-resistance in pancreatic cancer (84)	Higher SQLE associated with poor OS (20)
Colorectal cancer	SQLE overexpression is associated with lymphovascular invasion, tumor budding, advanced pT stage, and regional lymph node metastasis (63)	No relevant research	Higher SQLE associated with poor OS in patients with stage II, III tumor Lower SQLE predicts poor prognosis in T4 or stage IV tumors (63)
Lung squamous cell cancer	SQLE overexpression is associated with poor differentiation, TNM stage and lymph nodes metastasis (30, 61)	No relevant research	Overexpression of SQLE protein/mRNA is associated with poor OS (30, 61)
Uveal melanoma	No relevant research	No relevant research	Higher SQLE/mRNA is associated with poor OS, PFS and DFS (24, 85)
Nasopharyngeal cancer	No relevant research	No relevant research	Higher SQLE is associated with poor OS (13)
Head and neck squamous cell cancer	SQLE associated with T stage, tumor microenvironment (24)	No relevant research	Higher SQLE is associated with poor OS and PFS (24)
leukemia	No relevant research	Higher SQLE in Daunorubicin-resistant leukemia cells (25)	Higher SQLE predicts poor OS and EFS (104)

In prostate cancers, SQLE, which is involved in hormonal signaling, is associated with a high Gleason score, which implies poor biological behavior and prognosis of tumors (71, 72). After Gleason score matching, the expression of SQLE is higher in high-grade acinar cancer than in ductal carcinoma of the prostate, an unusual subtype of prostate cancer (73). This may imply that the role SQLE plays is different in different subtypes of cancer. High expression of SQLE was reported to be associated with poor outcomes in several case–control studies (72, 74). Higher SQLE is associated with metastasis (P = 1.1e-08, HR=3.7 [2.3−5.9]) in prostate tumors (65), and SQLE can predict metastasis combined with TPD52L2 (75). In locally advanced prostate cancer, the expression of SQLE is negatively associated with progression-free survival (PFS) (rs=-0.40) (76). Of note, both the primary tumor lesion and metastatic lesion overexpressed SQLE, which suggested that SQLE is involved in tumor growth and tumor metastasis (65). Androgen deprivation therapy (ADT) is the gold standard for hormone-sensitive metastatic prostate cancer (mHSPC) (77). However, mHSPC eventually develops into metastatic castration-resistant prostate cancer (mCRPC), which indicates poor prognosis after ADT. Overexpression of SQLE is involved in the resistance to castration mediated by metabolic reprogramming. Knockdown of SQLE can reverse this resistance (78).

In breast cancers, SQLE overexpression often indicates a more aggressive tumor (17) and is associated with tumor recurrence and short overall survival time, including estrogen receptor-positive (ER(+)) breast cancer and estrogen receptor-negative breast cancer or luminal A subtype and luminal B subtype (14, 16, 79, 80). Consistently, as early as 2007, it was reported that the amplification of 8q24.11-13 (regions including the SQLE gene) was associated with worse prognosis (81). BROWN D N et al. pinpointed that this amplification was associated with SQLE overexpression (17). The level of SQLE can also predict the response to estrogen deprivation therapy in ER (+) breast cancer. Tumor patients with SQLE overexpression commonly exhibited poor response to letrozole (P=0.38) and poor PFS under adjuvant tamoxifen (P<0.001, HR=2.02, 95% CI=1.5-2.7) (15). Larger tumor size, advanced TNM stage, HER2(+) status and lymph node metastasis are also associated with SQLE overexpression (16, 17).

In HCC, overexpression of SQLE is associated with poor prognosis (82). Similarly, copy number gains on 8q24.13-24.3 (area containing the SQLE gene) also indicated poor survival (83). Multivariate Cox analysis also suggested that SQLE was an independent biomarker of overall survival (28). High SQLE expression was associated with advanced TNM stage (p=0.045) and a-fetoprotein elevation (p=0.029) (82). For pancreatic cancer, the clinical relevance of SQLE is limited. SQLE overexpression is associated with poor overall survival (20), but no evidence has shown that SQLE is an independent prognostic factor for pancreatic cancer (20, 64). SQLE is associated with radio resistance in pancreatic cancer cells, but the correlation is much weaker than that of FDPS and IDI1 (84).

For colorectal cancer, a higher level of SQLE is associated with lymphovascular invasion, tumor budding, advanced pathological T stage, and regional lymph node metastasis (63). However, the prognostic prediction effect of SQLE seems to shift during tumor progression. A higher level of SQLE in tumors is associated with worse overall survival in a cohort mainly containing stage II and III patients (63). However, lower SQLE expression in T4 or stage IV tumors predicts worse prognosis. For lung squamous cell carcinoma, SQLE is associated with poor survival and clinicopathological variables, including poor differentiation and lymph node metastasis (30, 61). SQLE overexpression is also associated with worse survival in uveal melanoma (85), nasopharyngeal carcinoma (13), and head and neck squamous cell carcinoma (24). Daunorubicin-resistant leukemia cells express higher levels of SQLE than daunorubicin-sensitive leukemia cells (25). SQLE is associated with immune cell infiltration in tumors in head and neck squamous cell carcinoma based on bioinformatics analysis (24).

In most tumors, a high SQLE level predicts poor prognosis (except T4 or stage IV colorectal cancer), including tumor recurrence, tumor metastasis, higher-grade clinicopathological variables, and short overall survival time. In addition, as the key enzyme in cholesterol (the precursor of sex hormones) synthesis, overexpression of SQLE is associated with a poor response to hormone therapy. Collectively, considering the role of SQLE in tumorigenesis and tumor progression evidenced by basic studies and clinical analysis, SQLE may be a novel target for cancer therapy.

## SQLE-targeted therapeutic strategies for cancer treatment

In the fungus, the inhibition of SQLE leads to a lack of ergosterol and the accumulation of squalene (86). Hence, SQLE inhibitors are widely used against fungal infections (87, 88). SQLE inhibitors can be classified into allylamines, squalene derivatives, natural compounds and derivatives according to their structure. Naftifine, the first antifungal agent and a representative allylamine inhibitor (89), paves the way for next-generation inhibitors: terbinafine, NB-598, Cmpd-4, FR194738, etc.

Given SQLE deregulation in cancers and its tumor promotion function, targeting SQLE is believed to be a novel and promising antitumor therapy. Allylamines, as pioneers of SQLE inhibitors, were investigated for antitumor therapy. In a retrospective cohort study, patients with prostate cancer receiving systemic use of terbinafine had a decreased risk of overall death (HR=0.64; 95% CI, 0.52–0.77) and a decreased risk of death from prostate cancer (HR, 0.64; 95% CI, 0.52–0.77), while the topical use of terbinafine seemed not to bring survival benefits (90). Recently, four patients with rapidly progressive end-stage metastatic prostate cancer after multiple prior treatment modalities, excluding ADT and radiation, were off-label administered terbinafine orally. A drop in prostate-specific antigen (PSA) levels in three of the four patients was observed, suggesting that SQLE blockade can reduce biochemical markers of disease progression in prostate cancer (41).

SQLE inhibitors (terbinafine and NB-598) have been confirmed to suppress cell proliferation, blunt cell viability, promote cell death *in vitro*, and retrain tumor growth *in vivo* across various types of cancers in a dosage-dependent manner (17, 22, 66). Retardation of the cell cycle at the G0/G1 phase and enhancement of apoptosis are involved in the antitumor function of terbinafine, while there is no impact on normal cells (91). For the arrest of the cell cycle at G0/G1 phase, cell cycle regulators exhibit corresponding changes: a decrease in the levels of CDK4, phosphorylation of Rb, CDK2, and the p53-activated signaling pathway is involved in terbinafine-induced cell cycle arrest (66, 91, 92). The evidence of apoptosis includes the increased expression of cleaved caspase-7 and cleaved caspase-9 and DNA strand breaks caused by endonuclease (13, 91).

The mechanism underlying the suppression of tumor growth by SQLE inhibitors was investigated in previous studies. In nonalcoholic fatty liver disease (NAFLD)-induced HCC, terbinafine enhanced the degradation of SQLE *via* autophagy and then reversed the expression of PTEN, which in turn inhibited AKT/mTOR signaling (28). Consistently, terbinafine can also downregulate AKT activity, probably by decreasing cholesteryl ester (13). In breast cancer cell lines, the inhibition of SQLE by terbinafine decreased the phosphorylation of ERK (59). The accumulation of squalene induced by SQLE inhibitors can also be toxic to tumor cells. The detrimental effect of squalene can be accentuated when the cholesterol biosynthetic flux is increased, while lipid droplets can constrict the toxic effects by storing squalene (68, 93). Squalene has been thought to be nontoxic to cells for quite a long time (94). This detrimental effect of squalene may largely depend on the metabolic status in tumors. This speculation was evidenced in a subset of lymphoma cells that lack SQLE, while squalene accumulation can prevent oxidative cell death (95).

It should be noted that the antitumor function of SQLE inhibitors might also be independent of SQLE. In HCC, terbinafine still exerts its anticancer function even after SQLE knockdown. Instead, terbinafine suppressed mTORC1 by activating AMPK in a SQLE-independent manner (66). In oral squamous cell carcinoma cells, terbinafine repressed cell growth by inhibiting the KSR1-Raf-MEK-ERK pathway, but the role of SQLE was not investigated in the study (67).

Preclinical studies have revealed the toxicity of SQLE inhibitors when used as antitumor agents. In small cell lung cancer, dogs and monkeys treated with allylamine inhibitors (NB-598 and cmpd-4’’) *via* oral gavage cannot tolerate predicted efficacious exposures, with dose-limiting toxicity due to dose-limiting gastrointestinal toxicity, accompanied by skin toxicity. When naftifine and terbinafine were used as antifungal agents, the reported adverse reaction profiles were similar to these preclinical toxicology profiles (96, 97). This toxicity might limit the potential therapeutic utility for cancer treatment (98). The IC50 values of allylamine inhibitors in mammalian cells are several orders of magnitude higher than those in fungi (2, 99, 100). With increasing dosage to achieve antitumor efficacy, the tolerability of adverse reactions needs to be assessed carefully. Insights into the structure of SQLE in complex with allylamines offer further understanding of the mechanism of inhibition and new drug development. For allylamines, the tertiary amine motif is a common feature that interacts with the hydroxyl moiety of Y195 in the catalytic domain to form a hydrogen bond and can explain its noncompetitive inhibition (5).

For other types of SQLE inhibitors, such as natural compounds and derivatives, their special properties may enable themselves to be potential antitumor agents or a starting point to develop clinically safe SQLE inhibitors. (-)-Epigallocatechin 3-O-gallate (EGCG) extracted from green tea has been proven to be a potent (IC50 = 0.69 μM) and safe SQLE inhibitor, and few side effects have been reported even when it is consumed at high doses (101). The antitumor effect of EGCG has been widely investigated (69, 70), but it is currently unknown whether the association between SQLE and EGCG contributes to the effect. Selenocystine (IC50 = 65 μM) and S-allylcysteine (IC50 = 110 μM), the components extracted from garlic (102), ellagitannins isolated from various plants (103), etc., might also be potent SQLE inhibitors.

Taken together, SQLE inhibitors are potential antitumor agents considering their antitumor effects in various cancers (**Table 2**). Diverse types of SQLE inhibitors offer different frameworks to develop new SQLE inhibitors to ablate side effects and improve affinity.

**Table 2:** SQLE-targeted therapies in cancer.

SQLE inhibitors	Clinical development for cancer treatment	Cancer type	Action	Reference
Allylamines				
terbinafine	Off-label use	Prostate cancer	PSA level drop in three of the four patients	(41)
	Preclinical development	NAFLD-induced HCC	repress the viability of cancer cell *via* SQLE autophagy/PTEN/AKT restrain the tumor growth	(28)
	Preclinical development	Nasopharyngeal cancer	Suppress cell growth *via* cholesteryl ester/AKT Enhance apoptosis-related genes Restrain tumor growth and improve the survival of mice	(13)
	Preclinical development	Breast cancer	Repress cell viability *via* SQLE/ERK	(59)
	Preclinical development	HCC	Repress cell proliferation *via* AMPK-mTOR (independent of SQLE)	(66)
	Preclinical development	oral squamous cell carcinoma	Reduce proliferation and induce apoptosis *via* KSR1-Raf-MEK-ERK	(67)
	Preclinical development	Colorectal cancer	repress the viability of cancer cell induce G0/G1 arrest inhibit tumor growth	(22)
NB-598	Preclinical development	neuroendocrine cancer	Inhibit cell growth *via* squalene accumulation Inhibit tumor growth	(68)
	Preclinical development	Colorectal cancer	Disrupt cell proliferation and cell cycle Inhibit tumor growth	(22)
natural compounds and derivatives				
EGCG	Preclinical development	Various cancers	Induce cell apoptosis, inhibit cell proliferation	(69, 70)

## Conclusion

As an oncogenic gene in various cancers, the dysregulation of SQLE correlates with suppressing apoptosis and increasing cell proliferation and aggressiveness. The high abundance of SQLE in tumors indicates worse prognosis. Therefore, SQLE seems to be an attractive target for novel anticancer therapy. Delightfully, an increasing number of preclinical studies have revealed the antitumor effects and related mechanisms. However, the SQLE inhibitors applied in preclinical antitumor studies are mainly terbinafine, which is less selective to human SQLE, and the toxicity may limit antitumor therapy. The insights of the SQLE structure offer further understanding of new drug development to reduce adverse reactions and improve selectivity. Moreover, the metabolic status of tumor cells needs to be investigated before the application of SQLE inhibitors.

## Author contributions

HX made contributions to the conception and design. YZ and HX wrote the manuscript. HZ and QT helped to revise the manuscript. FB helped to collect the data. All authors contributed to the article and approved the submitted version.

## Funding

This research was supported by the National Natural Science Foundation of China (82103364); the Sichuan Science and Technology Program (2022YFS0204); the Postdoctoral Innovative Talents Support Program of China (BX2021203); and the Sichuan University postdoctoral interdisciplinary Innovation Fund.

## Conflict of interest

The authors declare that the research was conducted in the absence of any commercial or financial relationships that could be construed as a potential conflict of interest.

## Publisher’s note

All claims expressed in this article are solely those of the authors and do not necessarily represent those of their affiliated organizations, or those of the publisher, the editors and the reviewers. Any product that may be evaluated in this article, or claim that may be made by its manufacturer, is not guaranteed or endorsed by the publisher.

## Glossary

3’UTR: 3’ untranslated region
ABCA1: ATP-binding cassette transporter A1
ADT: Androgen deprivation therapy;
AMPK: adenosine 5’-monophosphate (AMP)-activated protein kinase
CASIMO1: cancer-associated small integral membrane open reading frame 1
CYP24A1: cytochrome P450 family 24 subfamily A member 1
DNMT3A: DNA methyltransferase 3A;
EGCG: epigallocatechin-3-gallate
EMT: epithelialmesenchymal transition
ER(+): estrogen receptor-positive
ER: endoplasmic reticulum
ERAD: endoplasmic reticulumassociated degradation
ERK: extracellular signal-regulated kinase
GSK3b: glycogen synthase 3b
HCC: hepatocellular carcinoma
HMGCR: 3-hydroxy-3-methylglutaryl-CoA reductase
LncRNA: Long noncoding RNA
MARCH6: membrane-associated RING finger 6
mHSPC: hormonesensitive metastatic prostate cancer
miRNA: microRNA;
MVA: mevalonate
NAFLD: nonalcoholic fatty liver disease;
NF-Y: nuclear factor Y
OSBPL2: oxysterol binding protein like 2
PCBP2: poly(rC) binding protein 2
RE1: response element 1
S1P: site-1 protease
S2P: site-2 protease
SCAP: SREBP2 cleavage-activating protein
SOAT1/2: sterol Oacyltransferase;
Sp1: specificity protein 1
SQLE: squalene monooxygenase
SRE: sterol-regulatory elements
SREBP2: sterol regulatory element-binding protein 2
UBE2J2: E2 ubiquitin-conjugating enzyme J2
USFA: unsaturated fatty acids
VCP: Valosin-containing protein
